# A Bio-Inspired Data-Driven Hybrid Optimization Framework for Task Unit Partition in Cruise Itinerary Planning

**DOI:** 10.3390/biomimetics11040239

**Published:** 2026-04-02

**Authors:** Zixiang Zhang, Dening Song, Jinghua Li

**Affiliations:** 1 College of Shipbuilding Engineering, Harbin Engineering University, 145 Nantong Road, Harbin 150001, China; 2 College of Mechanical and Electrical Engineering, Harbin Engineering University, 145 Nantong Road, Harbin 150001, China

**Keywords:** cruise itinerary planning, bio-inspired algorithm, data-driven optimization, hybrid framework, task unit partition, multi-objective clustering

## Abstract

Personalized itinerary planning for large-scale passengers under resource constraints is a critical challenge in enhancing the operational efficiency and service quality of cruise tourism. Traditional clustering methods, which primarily rely on geometric similarity, often fail to address the intricate coupling between passenger preferences and finite venue capacities, lacking predictive capability for the ultimate planning quality. To overcome these limitations, this study proposes a novel bio-inspired data-driven hybrid optimization framework for the cruise itinerary planning task unit partition. The framework innovatively integrates a Genetic Balanced Clustering Algorithm (GBCA) for multi-objective passenger grouping, Kernel Principal Component Analysis (KPCA) for feature extraction from preference data, an improved Adaptive Spiral Flying Sparrow Search Algorithm (ASFSSA) for hyperparameter optimization, and a Kernel Extreme Learning Machine (KELM) for data-driven prediction of itinerary planning quality. This synergy enables the framework to dynamically allocate venue capacities based on group preferences and optimize partitioning towards maximizing overall benefits, ensuring load balance and fairness. Extensive experiments on simulated cruise scenarios demonstrate that the proposed framework significantly outperforms conventional methods, improving segmentation quality by at least 40% while exhibiting superior convergence speed and stability. This work provides a scalable, intelligent solution for complex resource-constrained scheduling problems, showcasing the effective application of bio-inspired data-driven methodologies in engineering optimization.

## 1. Introduction

The cruise tourism industry has experienced rapid growth in recent years [1], driven by increasing demand for personalized travel experiences and the rise of smart tourism technologies, with annual passenger volumes exceeding 34.6 million in 2024 [2]. This rapid expansion has intensified competition among cruise operators, requiring innovative approaches to improve service quality and operational efficiency. In this context, cruise operators are under pressure to improve service quality and competitiveness by providing passengers with tailored itinerary planning [3]. However, the unique environmental constraints of cruise ships, including limited venue capacity, fixed hours of operation and service configurations, result in existing multi-passenger itinerary planning methodologies facing significant challenges when accommodating thousands of passengers simultaneously. For instance, when applied to a typical large cruise ship scenario with over 200 passengers and 20+ service venues, traditional itinerary optimization algorithms may require hours of computation time, failing to provide real-time or near-real-time solutions [4]. Moreover, they often produce plans where popular venues like the main dining room or theater are severely overcrowded, while others remain underutilized, leading to widespread passenger dissatisfaction and operational bottlenecks. This renders them impractical for real-world operations [5].

To address this issue, a promising approach involves grouping passengers with similar preferences and then generating optimized itineraries for each group. This strategy reduces the problem scale by partitioning passengers into manageable task units before applying optimization algorithms, thereby mitigating the computational burden of large-scale itinerary planning. The effectiveness of this approach, however, hinges critically on the rationality of passenger grouping, which must account for multiple interrelated factors: heterogeneous passenger preferences, finite venue capacities, and individual time budgets.

Research on itinerary optimization has evolved from single-passenger planning using meta-heuristic techniques such as GRASP (Greedy Randomized Adaptive Search Procedure) [6] and evolutionary algorithms [7] to group-oriented methods incorporating environmental constraints [8] and capacity limitations [4]. Despite these advances, existing group itinerary optimization methods remain computationally prohibitive for large-scale cruise scenarios [4,8,9]. This computational bottleneck underscores the necessity of effective passenger grouping prior to itinerary generation—a task that is itself non-trivial due to its inherently complex nature. The task unit partition problem in cruise itinerary planning is characterized by multiple conflicting objectives (maximizing itinerary benefit, balancing group sizes, and ensuring benefit equity), nonlinear relationships between passenger preferences and venue capacity constraints, and an exponentially growing solution space as passenger numbers increase. A key challenge—and a central innovation of this work—is the need to incorporate a predictive model of itinerary planning quality directly into the partitioning objective. This means that the quality of a given passenger grouping must be evaluated not by a simple geometric metric (such as distance or similarity), but by a data-driven prediction of the itinerary benefits that group would ultimately achieve.

Clustering algorithms serve as fundamental tools for identifying passenger cohorts with homogeneous preferences. Among various paradigms, partition-based clustering [10] and density-based clustering [11] have demonstrated particular efficacy in user segmentation applications. The K-means algorithm, as a prominent representative of partition-based clustering [12], has been widely applied and subsequently improved through better initial centroid selection [13] and weighted distance metrics [14]. However, a critical challenge specific to cruise itinerary planning is the need to evaluate the quality of each task unit’s itinerary after partitioning. Conventional clustering algorithms rely on cluster validity indices—such as CH [15], DB [16], and Dunn [17] metrics—to determine optimal cluster numbers. Recent years have seen the development of improved validity metrics, including those based on morphological similarity distance [18], CSP for structured data [19], STR using inflection point detection [20], FCVI [21], and HCVI for visual tree learning [22]. Despite these advancements, all existing validity metrics share a fundamental limitation: they compute validity based on distance between passenger data vectors, failing to capture the specific requirements of task unit partition for cruise itinerary planning, such as predicted itinerary quality and balanced resource allocation.

Parallel to developments in clustering, significant advancements have been made in applying bio-inspired optimization algorithms to partitional clustering problems. Researchers have proposed hybrid frameworks combining Chicken Swarm Optimization with K-harmonic means [23], bat algorithm variants with neighborhood search mechanisms [24], multi-medoid ant colony optimization for automatic cluster number determination [25], black hole phenomenon-inspired algorithms [26], and firefly algorithm variants [27]. Hybrid approaches have further integrated genetic algorithms with artificial bee colony optimization [28], ABC combined with Extreme Learning Machines [29], magnetic force-based search [30], and gravitational neighborhood strategies [31]. Recent innovations address initialization challenges through cooperative evolution [24], greedy seed selection [32], and neighbor heuristic functions [33], while enhanced gray wolf optimization [34] and chemical reaction optimization [35] have achieved improvements in solution accuracy. However, despite demonstrating satisfactory performance in single-objective cluster formation (e.g., spatial proximity minimization), these heuristic clustering methods exhibit intrinsic limitations when applied to cruise itinerary planning. Their fitness functions do not incorporate itinerary planning quality prediction, and they lack mechanisms to balance the competing requirements of operational efficiency, passenger experience, and capacity constraints. Crucially, the absence of cruise-specific objective functions hinders concurrent optimization of passenger grouping and service resource partitioning, resulting in suboptimal solutions that fail to address the multi-objective nature of the problem.

Bio-inspired algorithms, when properly designed, offer a promising alternative to address these limitations. By emulating natural processes such as evolution, swarm behavior, and biological adaptation, they possess inherent capabilities for global search, population-based exploration, and most importantly, the ability to optimize toward arbitrary, non-differentiable objective functions. This makes them ideally suited to directly use a predictive model of itinerary quality as a fitness function, iteratively refining passenger groupings to maximize expected benefits, a task that traditional distance-based clustering methods cannot accomplish.

Consequently, a critical research gap emerges: existing clustering and itinerary planning methods lack an integrated framework capable of simultaneously addressing the tripartite challenge of (i) dynamically allocating constrained venue capacities based on group preferences, (ii) incorporating predictive assessment of itinerary quality into the partitioning objective, and (iii) achieving balanced multi-objective optimization that maximizes overall benefit, ensures load fairness, and maintains computational efficiency for large-scale, real-world cruise scenarios.

To bridge this gap, this study proposes a novel hybrid optimization framework—KPCA-ASFSSA-KELM-GBCA—for the cruise itinerary planning task unit partition. The framework seamlessly integrates four key components: a Genetic Balanced Clustering Algorithm (GBCA) for multi-objective passenger grouping guided by a composite fitness function; Kernel Principal Component Analysis (KPCA) for nonlinear feature extraction from preference data; an improved Adaptive Spiral Flying Sparrow Search Algorithm (ASFSSA) for hyperparameter optimization; and a Kernel Extreme Learning Machine (KELM) for data-driven prediction of itinerary planning quality. This integration enables the framework to dynamically allocate venue capacities based on group preferences and iteratively refine partitions toward maximizing overall benefits while ensuring load balance and fairness.

The contributions of this work are summarized as follows:

- Multi-Objective Task Unit Partition: Unlike traditional clustering methods that rely solely on geometric similarity, the proposed model incorporates three key objectives: ① maximize the average predicted benefit of passenger groups, ② balance the size of task units to ensure computational feasibility, and ③ minimize the difference in benefits between task units. This explicitly optimizes for operational efficiency and fairness, rather than just data compactness.
- Feature Extraction and Hybrid Optimization Prediction Framework: KPCA is applied to extract principal components from passenger preference data. An ASFSSA-KELM model is introduced to predict the itinerary planning quality. In contrast to standard machine learning models (e.g., SVM, BP) that use default or empirically set parameters, ASFSSA intelligently optimizes the kernel parameters and regularization coefficients of KELM. This combination improves the accuracy and robustness of prediction.
- Dynamic Venue Capacity Partitioning: A novel capacity partitioning mechanism is proposed. This differs from conventional approaches that treat venue capacity as a static, hard constraint for each group. Instead, our mechanism dynamically allocates capacity based on the collective preferences of each passenger group, ensuring that high-demand venues receive the appropriate resources while avoiding the problem of overcapacity.
- Comprehensive Experimental Validation: The proposed model is evaluated on a synthetic dataset simulating a real cruise ship scenario. Comparative experiments demonstrate that the model outperforms traditional clustering and optimization algorithms in terms of convergence speed, stability and overall planning quality.

This research is systematically organized into four sections to present a coherent structure. Section 1 introduces the research background, reviews the relevant literature to establish the research gap, and articulates the motivation and contributions of the proposed framework. The proposed hybrid optimization framework is introduced in Section 2, detailing its conceptual foundation and key components. Section 3 presents the experimental methodology and analyzes the obtained results. Finally, Section 4 concludes the study by summarizing the principal findings and suggesting potential directions for future investigations.

## 2. Materials and Methods

The fundamental challenge in cruise itinerary planning lies in resolving the inherent conflict between passengers’ heterogeneous demands and constrained service resources. While conventional clustering methods can group passengers by preference similarity, they fail to address three critical operational requirements: (1) dynamic capacity constraints, (2) predictive quality assessment of itinerary plans, and (3) multi-objective balanced optimization under real-world business conditions.

To overcome these limitations, we propose a hybrid optimization framework with three synergistic components:

- Predictive Modeling Phase

A task unit quality prediction model is developed through multi-source data fusion and feature extraction, translating multidimensional parameters (passenger preferences, venue capacities, and time budgets) into quantifiable planning benefit metrics.

- Dynamic Resource Allocation Mechanism

An adaptive global optimization module dynamically regulates task unit sizes and capacity distribution, preventing local resource overload while maintaining efficient service allocation across high-demand periods.

- Multi-Objective Evaluation System

The framework integrates three optimization criteria: maximization of average predicted benefit, balanced task unit scale distribution and minimization of inter-unit benefit disparity. This tripartite evaluation guides the algorithm toward globally optimal solutions that balance passenger satisfaction, computational efficiency, and equitable resource distribution.

**Figure 1:**
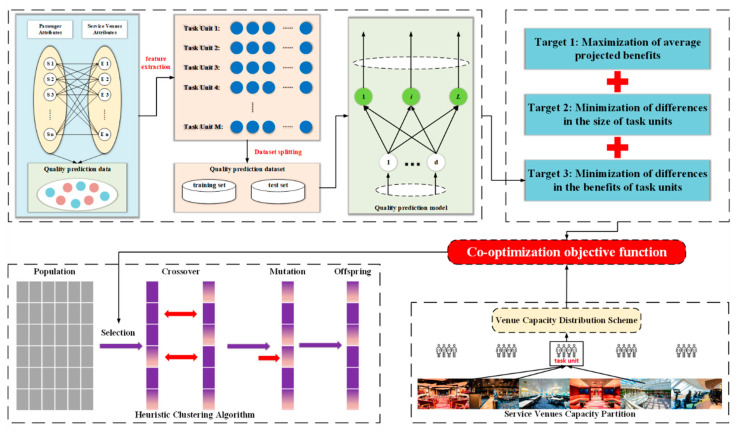
Architecture of the proposed hybrid optimization framework, illustrating the three core modules (predictive modeling, dynamic capacity allocation, and multi-objective evaluation) and their interconnections within the iterative optimization pipeline.

As illustrated in Figure 1, the proposed framework consists of three main modules working in synergy to generate balanced and high-benefit task unit partitions. The first module is a predictive modeling component based on KPCA-ASFSSA-KELM, which extracts key features from passenger preference data and predicts the expected itinerary quality for potential passenger groupings. The second module is a dynamic capacity allocation mechanism that adjusts venue capacity distribution in real-time based on group preferences and predicted demand, preventing local resource overload while maintaining service efficiency. The third module is a multi-objective evaluation system integrated into the Genetic Balanced Clustering Algorithm (GBCA), which simultaneously optimizes for average predicted benefit, balanced task unit scale distribution, and minimized inter-unit benefit disparity. These three components are interconnected in an iterative optimization pipeline: the predictive model informs the capacity allocation, which in turn guides the GBCA’s partitioning decisions, and the resulting partitions are fed back to refine the predictive model. This integrated architecture enables the framework to address the complex, multi-objective nature of cruise itinerary planning.

### 2.1. Venue Capacity Partition Based on Preference Data

Unlike the application scenario of conventional clustering methods, when constructing a cruise itinerary planning task unit, in addition to the partition of passengers, the capacity of cruise service venues needs to be split into individual task units to maximize the quality of itineraries ultimately obtained by all task units. Since passengers have different preferences for service venues, assigning higher capacity to service venues with higher preferences tends to yield more itinerary benefits for a task unit. Therefore, the capacity of the service venue for each task unit is determined by the average degree of preference for each service venue among all passengers in the task unit. The formula for calculating the service venue capacity for each task unit is as follows:

Unlike conventional clustering methods that partition only passengers, our framework also splits the capacity of each service venue among task units according to the collective preferences of the passengers within each unit. This ensures that high-demand venues receive proportionally more capacity, maximizing the potential itinerary benefit for each group.

Let $B_{m,i}$ denote the preference value of passenger m for service venue i. For a task unit z containing $M_{z}$ passengers, the average preference for venue i is

(1) $$P_{i,z}=\frac{\sum_{m=1}^{M_{z}}B_{m,i}}{M_{z}}$$

The initial capacity of venue i allocated to unit z is then computed as

(2) $$V_{i,z}=\left\lceil \frac{P_{i,z}}{\sum_{z=1}^{Z}P_{i,z}}\cdot C_{i}\right\rceil$$

where $C_{i}$ is the total capacity of venue i, and Z is the number of task units. Because of the ceiling operation, the total allocated capacity may exceed $C_{i}$. A correction step is therefore applied: for each venue, task units are sorted by increasing preference, and one unit of capacity is deducted from the least preferred units until the total matches $C_{i}$. The final allocated capacity is denoted as $V_{i,z}^{’}$.

### 2.2. Feature Engineering for Quality Prediction

To predict the itinerary planning quality of a task unit, we construct an 8-dimensional feature vector that captures both passenger characteristics and venue attributes. These features are designed to reflect the unit’s potential for generating high-quality itineraries. Table 1 lists the features and their meanings.

Features 7 and 8 are computed as follows. The overlap between the time window of service venue i and the time budget of passenger m is

(3) $${OTW}_{i,m}=\{\begin{array}{cc} min\left({SE}_{i},E_{m}\right)-max({SS}_{i},S_{m}),\;\; & E_{m}<{SS}_{i}\;or\;{SE}_{i}<S_{m}\\ 0,\;\; & else \end{array}$$

where $SS_{i},\;SE_{i}$ are the start and end times of venue i, and $S_{m},E_{m}$ are the start and end of passenger m’s time budget. The average effective service window length for task unit z is then

(4) $${AESWL}_{z}=\sum_{i=1}^{Q}\sum_{m=1}^{M}\frac{{OTW}_{i,m}}{Q}$$

where Q is the number of venues. The service venue capacity matching ratio is calculated as

(5) $${SVCMR}_{z}=\sum_{i=1}^{Q}\frac{V_{i,z}^{\prime }}{P_{i,z}}$$

This ratio measures how well the allocated capacity matches the group’s preferences, with higher values indicating better alignment. The target output for prediction is the total itinerary benefit obtained by applying a multi-passenger itinerary planning algorithm [4] to the task unit.

### 2.3. Predictive Modeling via KPCA-ASFSSA-KELM

To accurately predict the itinerary benefit from the eight features, we employ a Kernel Extreme Learning Machine (KELM) whose hyperparameters are optimized by an improved Adaptive Spiral Flying Sparrow Search Algorithm (ASFSSA). Before prediction, Kernel Principal Component Analysis (KPCA) reduces feature dimensionality and extracts nonlinear components.

**KPCA for Feature Extraction:** KPCA addresses the nonlinear relationships in passenger preference data by mapping the original feature vectors into a high-dimensional feature space via a nonlinear transformation ϕ, then performing linear PCA in that space. Given n samples, the kernel matrix K with elements $K_{ij}=k(x_{i},x_{j})$ is computed using the radial basis function (RBF) kernel:

(6) $$K\left(x_{i},x_{j}\right)=exp\left(-\frac{{\left\|x_{i}-x_{j}\right\|}^{2}}{2\gamma }\right)$$

The kernel matrix is centered to obtain $K^{*}=K-1_{n}K-K1_{n}+1_{n}K1_{n}$, where $1_{n}$ is an n×n matrix with all entries 1/n. Eigenvalue decomposition is performed on $K^{*}$, yielding eigenvalues $\lambda_{j}$ and eigenvectors $\eta_{j}$. The optimal number of retained features is determined based on the cumulative contribution rate, which is commonly set at 85% in dimensionality reduction tasks to balance information preservation and noise reduction [36]:

(7) $$\sum_{j=1}^{s}\lambda_{j}/\sum_{i=1}^{n}\lambda_{j}\geq 85\%$$

The original data are then projected onto the first s eigenvectors to obtain the reduced-dimensionality feature set $X^{t}$, which serves as input to the KELM predictor.

**KELM for Regression:** KELM improves upon the basic Extreme Learning Machine by replacing random hidden layer mappings with a kernel function, enhancing generalization and stability. The output function of KELM is:

(8) $$f_{KELM}(x)=\left[\begin{array}{c} k(x,x_{1})\\ \vdots \\ k(x,x_{N}) \end{array}\right]{\left(\frac{I}{C}+\Omega_{KELM}\right)}^{-1}T$$

where $\Omega_{KELM}$ is the kernel matrix with entries $\Omega_{ij}=k(x_{i},x_{j})$, C is the regularization parameter, I is the identity matrix, and T is the target output vector. The RBF kernel (Equation (6)) is used for its effectiveness in handling nonlinear relationships. The KELM’s performance critically depends on two hyperparameters: the regularization parameter C, which controls the trade-off between training error and model complexity, and the kernel width γ, which determines the influence range of each support vector.

**Figure 2:**
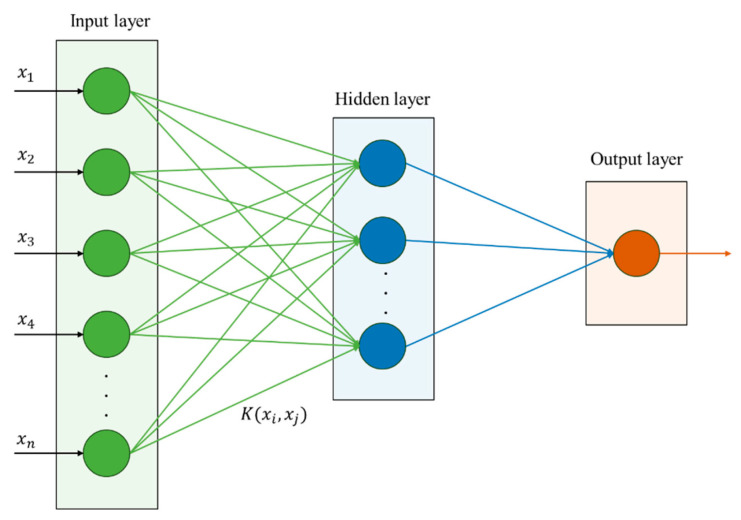
Structure diagram of KELM.

Figure 2 demonstrates that the KELM model employs the kernel trick, which obviates the requirement for prior knowledge regarding the number of hidden layer nodes, thus enhancing computational efficiency and model generalizability.

**ASFSSA for Hyperparameter Optimization:** To optimize C and γ, we employ an enhanced Adaptive Spiral Flying Sparrow Search Algorithm [37]. ASFSSA builds upon the basic Sparrow Search Algorithm by integrating four problem-specific enhancements:

- Tent chaotic mapping for population initialization, improving diversity and preventing premature convergence [38]. Its specific formula is as follows:

(9) $$z_{i+1}=\left(2z_{i}\right)mod1+rand(0,1)\times \frac{1}{N}$$

where N is the number of particles in the chaotic sequence.

- Adaptive weighting that dynamically adjusts the search intensity based on iteration progress, balancing exploration and exploitation [31]. The formula for adaptive weights is as follows:

(10) $$\omega \left(t\right)=0.2cos\left(\frac{\pi }{2}\left(1-\frac{t}{{iter}_{max}}\right)\right)$$

- Lévy flight mechanism enabling occasional long jumps to escape local optima [39]. The location update format for joining the Lévy flight strategy is as follows:

(11) $$x_{i}^{\prime }\left(t\right)=x_{i}\left(t\right)+l⨁levy(\lambda )$$

where $x_{i}\left(t\right)$ represents the position of the *i*-th individual in the *t*-th iteration, and ⨁ is an arithmetic symbol representing point-to-point multiplication. l denotes a step length control parameter. levy(λ) is a path that obeys the Lévy distribution.

- Variable spiral search strategy, inspired by whale hunting behavior, to refine solutions in promising regions [40].

**Figure 3:**
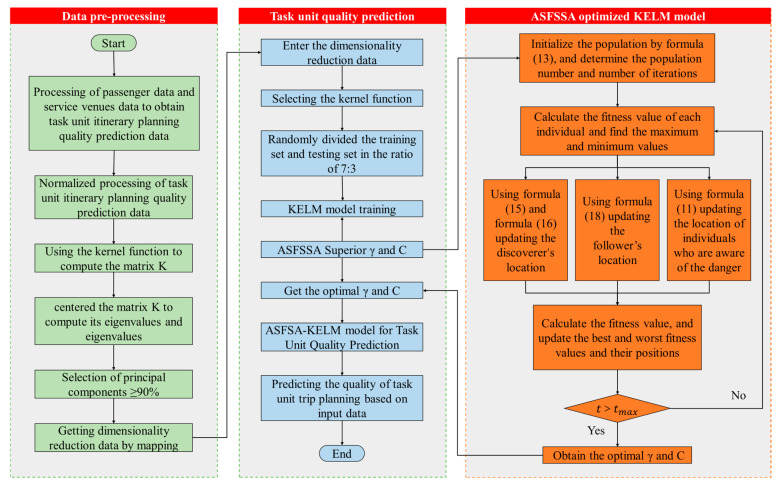
KPCA-ASFSA-KELM for task unit quality prediction.

The ASFSSA optimizes (C,γ) by minimizing the Root Mean Square Error (RMSE) on a validation set. The population consists of discoverers (responsible for global exploration), followers (who follow discoverers to promising regions), and warners (who perform local searches when danger is detected). Each agent updates its position according to adaptive rules that incorporate the above mechanisms. After optimization, the best-found (C,γ) are used to train the final KELM on the full training set. The complete predictive modeling pipeline is illustrated in Figure 3.

### 2.4. Genetic Balanced Clustering Algorithm (GBCA)

GBCA is a genetic algorithm specifically designed for multi-objective task unit partition. Unlike conventional clustering methods that rely on distance-based similarity metrics, GBCA directly optimizes partition quality using a composite fitness function that incorporates predictive itinerary benefit.

**Chromosome Representation and Initialization:** Each chromosome encodes a candidate partition of N passengers into Z task units. A population of P chromosomes is randomly initialized, ensuring diversity in the initial partitions.

**Fitness Evaluation:** The fitness of each chromosome is evaluated based on three normalized objectives:

Average Predicted Benefit (APB): The mean predicted itinerary benefit across all task units, obtained from the trained KPCA-ASFSSA-KELM predictor:

(12) $$APB=\sum_{z=1}^{Z}{PV}_{z}/Z$$

where $PV_{z}$ is the predicted benefit for task unit z.

**Size Relative Range (**${RR}_{s}$**):** The disparity in task unit sizes, normalized by the average size:

(13) $${RR}_{s}=max\left(\left|U_{z}\right|\right)-min\left(\left|U_{y}\right|\right)/\left|\bar{U}\right|$$

where $|U_{z}|$ is the size of task unit z and $|\bar{U}|$ is the mean unit size.

**Benefit Relative Range (**${RR}_{v}$**):** The disparity in predicted benefits among units, normalized similarly:

(14) $${RR}_{v}=max\left(\left|V_{z}\right|\right)-min\left(\left|V_{y}\right|\right)/\left|\bar{V}\right|$$

Specifically, the fitness function is formally defined as a weighted sum:

(15) $$F=min\left({{w_{1}\cdot Target}_{1}+w_{2}\cdot Target}_{2}+w_{3}\cdot {Target}_{3}\right)$$

where w1, w2, and w3 are the non-negative weights of the three sub-objectives. In this study, we adopt equal weights ($w_{1}=1,w_{2}=1,w_{3}=6$) as a baseline configuration to provide a balanced trade-off among the three objectives. This equal-weight setting serves as a neutral starting point in the absence of specific business preferences.

Importantly, the framework is designed with flexibility in mind: cruise operators can readily adjust these weight coefficients to reflect their strategic priorities. For example, an operator aiming to maximize overall passenger satisfaction might increase $w_{1}$, while another prioritizing fairness across groups could assign higher values to $w_{2}$ and $w_{3}$. This adaptability ensures that the proposed approach can be tailored to diverse real-world operational requirements.

**Genetic operators:** GBCA employs the following:

- Tournament selection to choose parents for reproduction, maintaining selection pressure while preserving diversity.
- Uniform crossover with probability $p_{c}=0.8$ to exchange genetic material between parents.
- Mutation with probability $p_{m}=0.2$ that randomly reassigns a small number of passengers to different task units, introducing novel genetic material.

**Termination:** The evolution proceeds for a maximum of $T_{max}=100$ generations or until the fitness improvement over 20 consecutive generations falls below a threshold of $10^{-4}$. The chromosome with the best fitness at termination is output as the optimal task unit partition.

### 2.5. Integrated Framework Workflow

**Figure 4:**
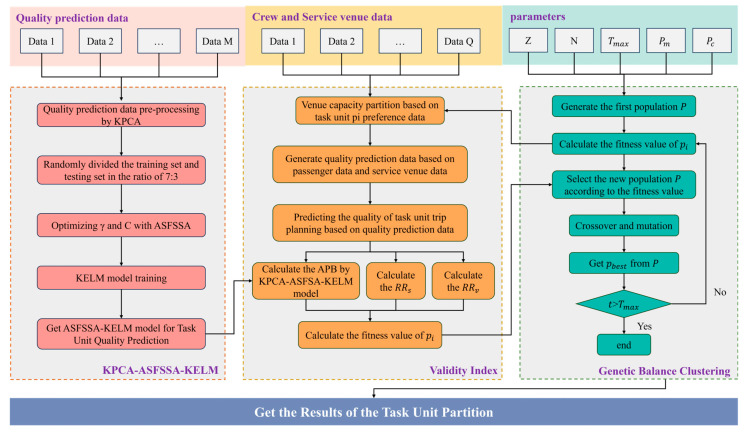
The workflow diagram of the KPCA-ASFSSA-KELM-GBCA.

The complete KPCA-ASFSSA-KELM-GBCA framework operates through the following integrated workflow (Figure 4):

- **Offline Training Phase:** Using historical or synthetic task unit data, the KPCA-ASFSSA-KELM predictor is trained to map the eight engineered features to the corresponding itinerary benefit. This trained predictor serves as the fitness oracle for the subsequent clustering phase.
- **Initialization Phase:** For a given set of passengers and venues, GBCA generates an initial population of random partitions, each assigning passengers to Z task units (where Z is determined via the elbow method described in Section 3.3).
- **Capacity Allocation Phase:** For each candidate partition, venue capacities are dynamically allocated to task units using the mechanism described in Section 2.1. This produces the final allocated capacities $V_{i,z}^{\prime }$ needed for feature computation.
- **Fitness Evaluation Phase:** For each task unit in the partition, the eight features are computed (Section 2.2) and fed into the trained predictor to estimate the benefit $PV_{z}$. The three objectives APB, $RR_{s}$, and $RR_{v}$ are then calculated, yielding the composite fitness F.
- **Evolutionary Optimization Phase:** Genetic operators (selection, crossover, mutation) are applied to generate a new population of partitions. Steps 3–4 are repeated for each generation, progressively improving the fitness of the population.
- **Termination and Output Phase:** Upon meeting termination criteria, the partition with the best fitness is output as the optimal task unit grouping. This partition simultaneously maximizes predicted itinerary benefit, balances task unit sizes, and ensures equitable benefit distribution.

This closed-loop integration of predictive modeling, dynamic resource allocation, and multi-objective evolutionary optimization enables the framework to discover partitions that are superior not only in feature-space metrics but also in terms of downstream itinerary planning performance, a capability that conventional clustering methods fundamentally lack.

## 3. Results

### 3.1. Datasets and the Experimental Environment

For the case study, we need to randomly generate the cruise itinerary planning task unit partition data and task unit quality prediction training dataset. Firstly, we randomly generate the passenger data and service venue data involved in the cruise itinerary planning task unit partition. To verify the performance of the algorithm on datasets of different sizes, we conduct experiments using datasets of different passenger sizes and service venue sizes, and the characteristics are detailed in Table 2.

The dataset sizes were chosen to reflect realistic cruise scenarios: Instance 1 corresponds to a small cruise ship with limited venues, while Instance 4 simulates a large vessel with more complex service offerings. These settings ensure the evaluation covers a wide range of operational scales.

All simulations assumed a fixed passenger origin at coordinates (0, 0, 0). Key parameters were randomly generated within specified ranges:

- Daily time budget constraints: start times [360, 540] minutes (6:00–9:00 a.m.);
- Activity windows: end times [1200, 1440] minutes (8:00 p.m.–12:00 p.m.);
- Service venue utility values: [1, 20] benefit units.

Time windows for each venue were standardized to a minute-based representation (e.g., 7:00 a.m. = 420 min) to ensure computational consistency in scheduling and overlap calculations. Service venue utility values are expressed as dimensionless benefit units within the range [1, 20], where higher values indicate greater passenger preference or perceived satisfaction. The complete parameter specifications for synthetic data generation are presented in Table 3.

The training dataset for quality prediction is then randomly generated for the partition of task units. The size of the training dataset is set to 3000, and each piece of data for each task unit is randomly generated in the interval. Where the interval of the number of passengers is [10, 80] and the default of passenger location is (0, 0, 0). The number of service venues interval is [10, 60], the location of service venues is randomly generated in the range of 100 × 100 × 100, and the capacity of service venues interval is [1, 30]. The generation intervals for service venue benefit values, recommended service hours, passenger time budgets, and service venue opening hours are the same as the previous generation intervals. Based on the passenger data and service venue data generated for each task unit, the quality prediction input data are fitted to form the quality prediction input data in the manner described in Section 2.2, and the multi-passenger itinerary planning algorithm [4] is applied to calculate the objective function values. These are used as input data and output data for the training dataset of quality prediction for the cruise itinerary planning task unit partition, respectively.

While the data generation process was carefully designed based on real-world cruise parameters (Table 3) to ensure a degree of realism, it is important to acknowledge the limitations of using fully synthetic data. The random generation may not fully capture the nuanced patterns and stochastic nature of actual passenger behavior or operational disturbances. Consequently, the reported performance improvements, while significant in these controlled synthetic settings, should be interpreted as a strong indicator of the framework’s potential. Validation on real-world cruise data is a crucial direction for future work.

All experiments were conducted on a desktop computer with the following specifications: 11th Gen Intel Core i7-11390H processor (3.40 GHz, 4 cores) (Intel Corp., Santa Clara, CA, USA), 16 GB RAM, running Windows 11. The proposed framework and all comparison algorithms were implemented in MATLAB R2022b, utilizing the Statistics and Machine Learning Toolbox for clustering algorithms. To ensure fair comparison, all methods were executed under the same experimental conditions and software environment.

### 3.2. Performance Evaluation of Predictive Model

The validity of the KPCA-ASFSSA-KELM model needs to be validated to ensure the accuracy of the quality assessment of the cruise ship itinerary planning task unit partition. We assessed the validity of the prediction model by analyzing the performance of KPCA-ASFSSA-KELM on a sample dataset of passenger itinerary planning quality predictions.

#### 3.2.1. Feature Extraction

The feature vector $X=\left\{x_{1},x_{2},\ldots ,x_{n}\right\}$ for cruise itinerary planning task unit quality Prediction comprises eight variables (Table 1). Prior to analysis, the dataset is normalized to constrain all variables within the range [0, 1]. To address potential feature interdependence and eliminate noise from irrelevant variables, Kernel Principal Component Analysis (KPCA) is applied for dimensionality reduction on the normalized feature vectors.

**Figure 5:**
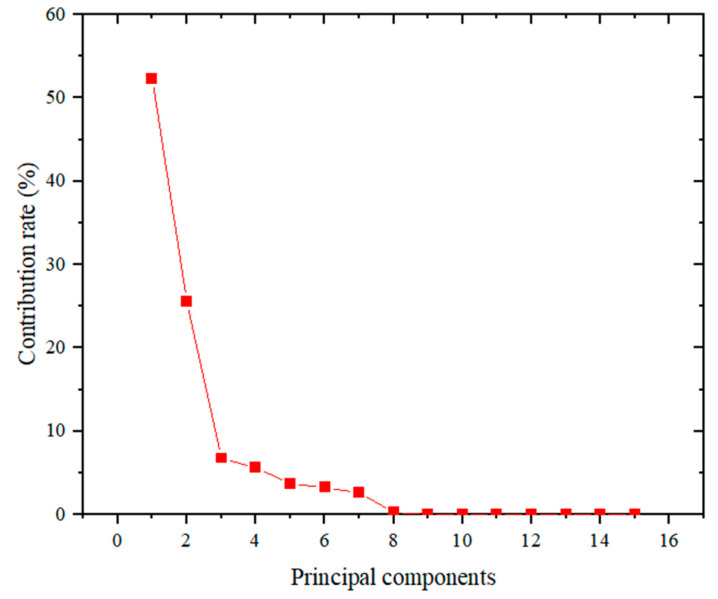
Contribution rate of each principal component.

Figure 5 presents the contribution rates of the principal components following dimensionality reduction. To identify the most informative components, a cumulative contribution rate threshold of 90% was applied. Table 4 summarizes the eigenvalues, individual contribution rates, and cumulative contribution rates of the top five principal components derived from KPCA. The results indicate that the first four principal components collectively account for over 90% of the cumulative contribution rates, effectively capturing the essential structure of the original dataset.

#### 3.2.2. Evaluation Criteria

To rigorously evaluate the predictive capability of the proposed model, four established evaluation metrics were employed:

Root Mean Square Error (RMSE), quantifying prediction accuracy:

(16) $$RMSE=\sqrt{\frac{1}{N}\sum_{n=1}^{N}\;(y\prime \left(n\right)-y(n){)}^{2}}$$

Mean Absolute Percentage Error (MAPE), assessing the percentage-based error magnitude:

(17) $$MAPE=\sqrt{\frac{1}{N}\sum_{n=1}^{N}\;|\frac{y\prime \left(n\right)-y(n)}{y(n)}|}\times 100\%$$

Mean Absolute Error (MAE), assessing absolute deviation:

(18) $$MAE=\frac{1}{N}\sum_{n=1}^{N}\left|y\prime \left(n\right)-y(n)\right|$$

Coefficient of Determination ($R^{2}$), evaluating goodness-of-fit:

(19) $$R^{2}=1-\frac{\sum_{n=1}^{N}{\left(y\left(n\right)-y\prime \left(n\right)\right)}^{2}}{\sum_{n=1}^{N}{\left(y\left(n\right)-\bar{y}(n)\right)}^{2}}$$

where y(n) and y′(n) refer to the actual measurement and prediction values. $\bar{y}(n)$ denotes the actual average value of the predicted object. N denotes the amount of data.

#### 3.2.3. Predictive Performance Evaluation and Comparative Analysis

The performance validation of the predictive model used 3000 sets of samples for simulation experiments. The four principal components after KPCA dimensionality reduction were used as the input set of the ASFSSA-KELM model, and the corresponding itinerary benefits of the cruise itinerary planning task unit were used as the output set. From the reconstructed dataset, 2400 groups are randomly selected as the training set of the model, and the remaining 600 groups are used as the test set. ASFSSA-KELM is compared with the other seven methods, namely, VPPSO-KELM [41], SSA-KELM [42], GWO-KELM [43], KELM [44], ELM [45], BP [46], and LSTM [47]. Each model is repeatedly run 20 times. The comprehensive prediction results, including performance metrics and error analysis, are presented in the following figures and tables. These quantitative outcomes facilitate an in-depth comparative evaluation of the model’s predictive capabilities.

**Figure 6:**
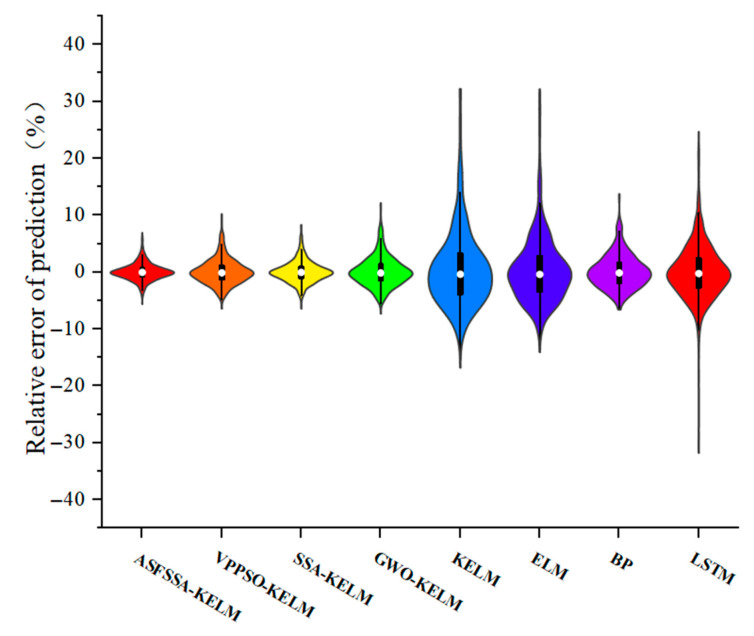
Relative error violin plots for each model.

Figure 6 displays the violin plot representation of prediction relative errors across all models. The interquartile range (IQR), visualized by the box length, serves as an indicator of error dispersion—with greater length corresponding to increased variability. Comparative analysis reveals the ASFSSA-KELM model exhibits the most constrained IQR, with error distributions tightly clustered near zero. This characteristic demonstrates superior error concentration compared to alternative approaches, suggesting enhanced prediction stability.

**Figure 7:**
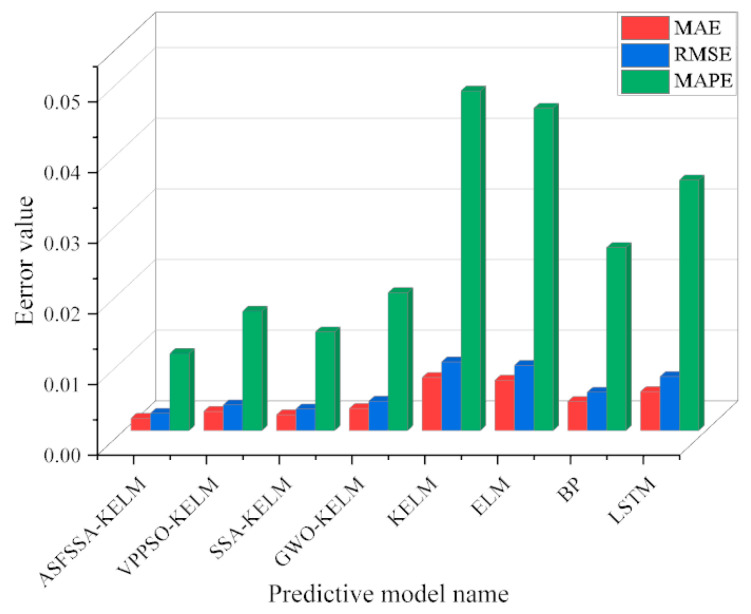
Comparison of MAE, RMSE and MAPE results for each model.

As detailed in Table 5, the proposed ASFSSA-KELM model demonstrates superior predictive performance across all evaluation metrics, achieving optimal values for RMSE (0.0024), MAE (0.0017), MAPE (1.09%), and $R^{2}$ (0.9886). The comparative histogram in Figure 7 consolidates MAE, RMSE, and MAPE results, visually confirming that the ASFSSA-KELM model yields the lowest error metrics among all evaluated methods.

**Figure 8:**
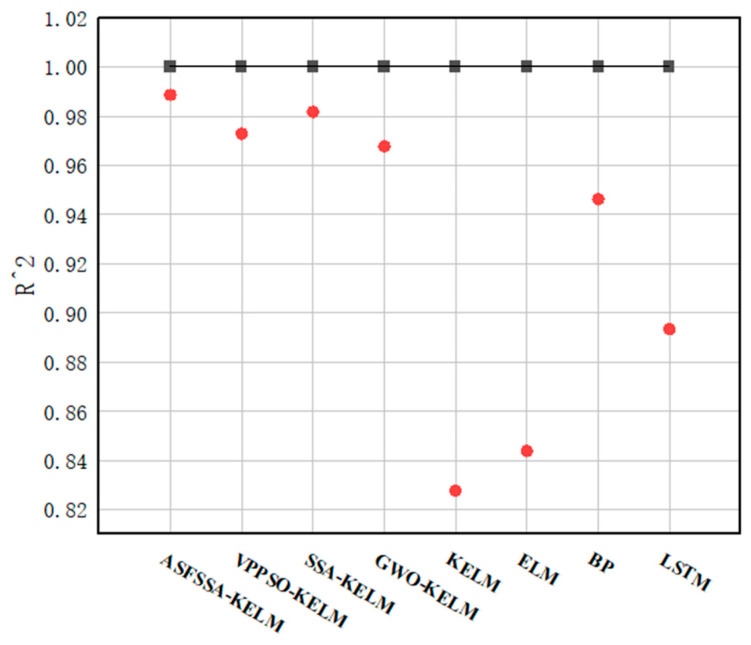
Comparison of $R^{2}$ results for each model.

It is worth noting that the $R^{2}$ values reported in Figure 8 span a wide range, from 0.8277 of KELM to 0.9886 of ASFSSA-KELM. The majority of models, including ASFSSA-KELM, SSA-KELM, VPPSO-KELM, GWO-KELM, and BP, achieve $R^{2}$ values above 0.94, indicating strong predictive performance. The relatively lower $R^{2}$ values observed for KELM, ELM, and LSTM are expected, because these models lack systematic parameter optimization or are not well-suited to the tabular structure of our prediction task. We include these weaker baselines intentionally: they provide a reference point that underscores the necessity of the proposed optimization framework, and highlight the substantial improvements achieved by our enhanced models.

The improvements achieved by the different prediction models across the four evaluation metrics are shown in Table 6, Table 7, Table 8 and Table 9. The experimental results demonstrate that the GWO-optimized KELM exhibits relatively inferior accuracy. However, the GWO-KELM hybrid achieves significant improvements over the baseline KELM model, with error reductions of 57.11% (RMSE), 59.39% (MAPE), and 56.17% (MAE), along with a 16.91% enhancement in $R^{2}$. These metrics collectively validate the superior parameter optimization capability of intelligent algorithms for KELM models.

Among conventional prediction approaches, the BP neural network yields the best performance. Notably, the proposed ASFSSA-KELM model outperforms BP by substantial margins: 55.13% lower RMSE, 57.92% reduced MAPE, 57.71% decreased MAE, and 4.48% higher $R^{2}$. This comprehensive superiority confirms that the ASFSSA-KELM architecture delivers markedly enhanced predictive accuracy compared to standard machine learning models.

Therefore, the KPCA-ASFSSA-KELM prediction model proposed in this paper possesses sufficient accuracy to accurately predict the itinerary planning quality of passenger groups. It can be used as a clustering validity indicator for passenger clustering grouping.

### 3.3. Determination of the Optimal Number of Task Units

To validate the effectiveness of the proposed model in performing the task unit partition for cruise ship itinerary planning, we tested it on the synthesized dataset described in Section 3.1. To obtain the optimal number of partitions, KPCA-ASFSSA-KELM-GBCA is run iteratively on the dataset to obtain the experimental results. *k*-values are set from 2 to $\sqrt{M}$, according to the commonly used rule [48], and M is the number of passengers in the dataset. The method used to determine the optimal number of task units is shown in the pseudocode in the Table 10.

**Figure 9:**
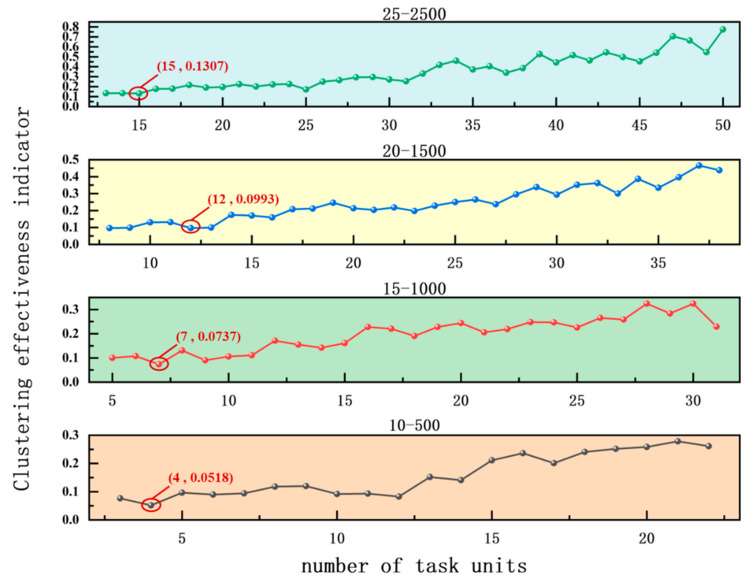
Experimental results on the optimal number of task units.

The data described in Section 3.1 are four simulated datasets. The experiment results of the optimal number of task units are shown in Figure 9. When task units are partitioned, exceeding a certain size can result in excessive time spent on itinerary planning. Therefore, when conducting experiments to determine the optimal number of task units, the upper limit of the average size of task units is set to 200. Since the objective function is a minimal value function, when this metric takes the minimum value, it indicates that the algorithm obtains the optimal partition of task units. As can be seen in the figure, the optimal number of task units for the four size datasets is 4, 7, 12, and 15, respectively.

### 3.4. Comparison of Algorithms for Task Unit Partition

Based on the determination of the optimal number of task units for the simulated dataset, and in order to validate the performance of the proposed GBCA, we conducted tests on the four types of datasets described in Section 3.1. The computed results are compared with seven other algorithms applied to the clustering task, including three optimization algorithms, ASFSSA [37], PSO [41], and BWO [49], and four clustering algorithms, K-means [12], K-medoids [50], SC [51], and K-means++ [52]. The user-defined parameters setting of GBCA are defined as the number of task units = $k_{opt}$ (obtained through the optimal number of task units experiment), population size = 100, crossover probability = 0.8, variation probability = 0.2. For comparison, we run each algorithm 30 times independently, taking the average value as the final result.

Table 11 presents the comparative results of eight algorithms on the simulated datasets in terms of average predicted benefit (APB), Relative Range of Task Unit Size (${RR}_{s}$), Relative Range of Task Unit Benefit (${RR}_{v}$), and the composite objective function (F). In addition, the standard deviation of F (SD(F)) is reported to evaluate the stability of each algorithm.

The results clearly demonstrate the efficacy of the proposed GBCA. From Instance 1 to Instance 3, GBCA consistently achieves the best values across all evaluation metrics, indicating its superior ability to balance itinerary benefit, size equilibrium, and benefit fairness. Specifically, GBCA obtains the lowest F values (0.0389, 0.1074, and 0.1587) on these datasets, significantly outperforming all compared methods. Moreover, the SD(F) of GBCA is the smallest among all algorithms on each instance, demonstrating its strong robustness and stability across independent runs.

When the dataset scale is further expanded to Instance 4, PSO achieves a marginally better APB (−0.1661) than GBCA (−0.1658). However, the difference is negligible, and GBCA still maintains the best overall performance in terms of the composite F value (0.1545), along with the lowest SD(F) (0.0076), indicating that it not only balances size and benefit effectively but also does so with high consistency. In contrast, traditional clustering algorithms such as K-means and K-medoids exhibit much larger SD(F) values, reflecting their sensitivity to initialization and lack of optimization guidance.

These results confirm that GBCA achieves a superior trade-off among multiple objectives and exhibits significantly better stability than both heuristic and conventional clustering methods, making it particularly suitable for large-scale, real-world cruise itinerary planning tasks.

To assess the statistical significance of the observed performance differences, we performed the Wilcoxon signed-rank test to compare the F-values obtained by the proposed GBCA against those of the seven competing algorithms across 30 independent runs. All tests were conducted at the 0.05 significance level (α=0.05). The test results confirm that the performance improvements achieved by GBCA over all comparison algorithms are statistically significant: all *p*-values are consistently below 0.01 across all four dataset instances. In our experiments, the performance differences between GBCA and every compared algorithm are uniformly negative (all sign bits are consistent); thus, all pairwise comparisons yield the identical *p*-value of $1.862645\times 10^{-9}$. This result robustly verifies that the superior performance of GBCA is not merely due to random chance.

**Figure 10:**
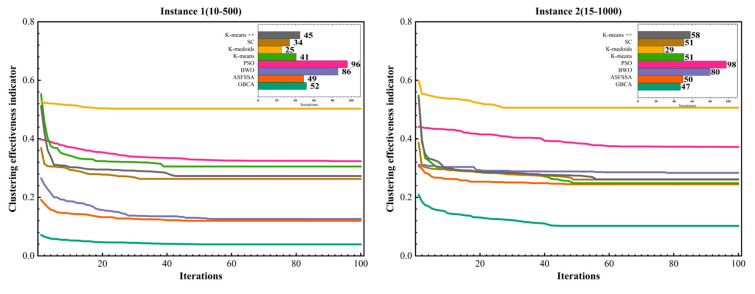

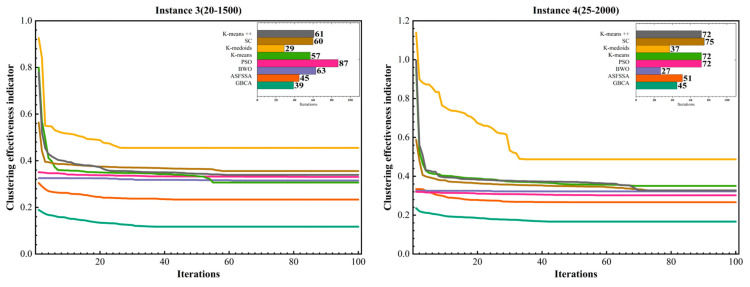
Convergence behavior and iteration counts of clustering algorithms.

Figure 10 shows the convergence behavior of GBCA, ASFSSA, BWO, PSO, K-means, K-medoids, SC, and K-means++ clustering algorithms. The main plots depict the objective function value versus the number of iterations for datasets of four different sizes, while the top-right bar chart compares the number of iterations each algorithm requires to reach convergence (defined as the point where the objective function value stabilizes). As can be seen from the graphs, the proposed GBCA exhibits good convergence speed on all datasets, reaching a steady state in about 40 iterations. Compared with other heuristic algorithms (e.g., PSO, BWO, ASFSSA), GBCA achieves a competitive convergence rate, often requiring fewer iterations than PSO and BWO, as illustrated by the bar chart, while its convergence curve is smoother and decreases at a uniform rate. This indicates that its optimization mechanism efficiently adapts to increasing data size.

Although some traditional clustering methods converge faster in terms of iteration number, they are extremely sensitive to the initial cluster center, so their convergence curves fluctuate greatly on datasets of different sizes, and they stop updating the partition result early after converging, without further optimization of the objective function, which makes them easily fall into local optima, and the final clustering effectiveness indicator is much worse than GBCA. In contrast, GBCA can continuously improve clustering quality during the iteration process and has obvious advantages in optimizing the objective function, delivering more stable and better clustering results.

Table 12, Table 13, Table 14 and Table 15 show the improvement of the proposed algorithm over the comparison algorithm in terms of the objective function and individual metrics. It can be seen that the GBCA improves F by 63.16%, APB by 0.89%, ${RR}_{s}$ by 56.67% and ${RR}_{v}$ by 33.64% compared with the comparison algorithms on average. In terms of individual indicators, the average predicted benefits of task units calculated by the comparison algorithm and GBCA are closer, but the performance difference between the heuristic clustering algorithm and the traditional clustering algorithm is significant in terms of the indicators of scale equalization and benefit value equalization.

As can be seen in these tables, the traditional clustering algorithm takes distance minimization as the core objective and directly constrains the sample partition through iterative optimization, which tends to generate clusters of similar sizes and performs better in the size equilibrium indicator. The heuristic algorithm supports explicit constraints through global random search and thus performs better in the benefit value equalization indicator. The GBCA combines the advantages of global optimization search and the weighted objective function of the heuristic algorithm, and sets explicit constraints for size equalization and benefit value equalization to avoid degradation of the equalization indicator. It can effectively solve the needs of business scenarios of task unit partition and realize scale stability and benefit balance maximization.

## 4. Discussion

To address the multi-objective optimization challenge in cruise itinerary planning, this study proposes a novel hybrid optimization framework integrating Genetic Balanced Clustering Algorithm (GBCA), Kernel Principal Component Analysis (KPCA), Adaptive Spiral Flying Sparrow Search Algorithm (ASFSSA), and Kernel Extreme Learning Machine (KELM). The framework initiates with KPCA for nonlinear feature extraction from passenger preference data, followed by a dynamic mechanism that allocates venue capacities based on group preferences. The core predictive capability is provided by a KELM model, whose parameters are optimized using the bio-inspired ASFSSA metaheuristic. The predicted itinerary quality is then embedded into a composite fitness function to guide the GBCA in iteratively refining the task unit partition, balancing benefit maximization, load fairness, and unit size equilibrium.

The proposed framework was rigorously evaluated through comprehensive experiments conducted on simulated datasets of varying scales (500 to 2500 passengers, 10 to 25 venues). A systematic performance comparison was performed against seven established clustering algorithms, including ASFSSA, PSO, BWO, K-means, K-medoids, SC, and K-means++. The experimental results demonstrate that the proposed framework achieves superior performance both quantitatively and statistically. Specifically, GBCA improves the composite fitness F by an average of 63.16% across all datasets compared to the seven baseline algorithms, with the most significant improvement reaching 92.73% on Instance 1 against K-medoids. In terms of individual objectives, GBCA achieves 0.89% higher average predicted benefit (APB), 56.67% better task unit size equalization (${RR}_{s}$), and 33.64% improvement in benefit equalization (${RR}_{v}$) compared to the baseline average. Furthermore, the Wilcoxon signed-rank test confirms that all performance improvements are statistically significant, indicating that the observed advantages are not attributable to random variation. Convergence analysis further reveals that GBCA reaches a steady state within approximately 40 iterations, demonstrating a competitive convergence rate while maintaining smoother and more stable convergence curves than heuristic alternatives.

In summary, this research presents a cohesive methodology that effectively couples data-driven prediction with bio-inspired optimization. The proposed framework demonstrates a scalable and intelligent approach to resolving the conflict between personalized service and constrained resources in complex scheduling problems, showcasing the practical synergy of bio-inspired data-driven methodologies in engineering optimization.

Finally, it should be noted that the current study is based on synthetic data; while the generation process was grounded in realistic cruise parameters, future validation of the model’s generalizability in real-world cruise operation scenarios is needed to confirm its practical efficacy and robustness under actual operational conditions. Future work could also explore adaptations of deep clustering methods to the task unit partition problem, potentially by integrating the predictive and capacity allocation modules proposed in this study into their frameworks. Additionally, further research should investigate reinforcement learning for real-time task unit adjustment and collaborative multi-ship planning under sustainability constraints. This work provides theoretical support and technical solutions for large-scale resource allocation problems in smart tourism, and its potential applications can be extended to other domains that require multi-objective optimization in constrained environments.

## Figures and Tables

**Table 1:** Data items and meaning of quality prediction data.

No.	Feature	Description
1	Number of Passengers	Total number of passengers in the task unit
2	Average Passenger Time Budget	Mean value of budgeted hours of passenger time in the task unit
3	Average Passenger Preference Value	Mean value of passenger preference for each service venue in the task unit
4	Number of Service Venues	Total number of service venues in the task unit
5	Total Capacity of Service Venues	Total number of capacities of all service venues in the task unit
6	Average Recommended Service Length	Average of recommended service hours for all service venues in the task unit
7	Average Effective Service Window Length	Average of the sum of the overlap between the hours of operation of each service venue and the time budget of the passengers in the task unit
8	Service Venue Capacity Matching Ratio	Average of the ratio of each passenger’s preference for each service venue to the equipped capacity of that service venue in the task unit

**Table 2:** Characterization of datasets.

Datasets	D (Number of Service Venues)	N (Number of Passengers)
Instance 1	10	500
Instance 2	15	1000
Instance 3	20	1500
Instance 4	25	2500

**Table 3:** Generation of data on cruise ship service venues.

Type	1	2	3	4
Starting time interval	[360, 540]	[720, 810]	[720, 810]	[0, 480]
Ending time interval	[690, 780]	[1020, 1110]	[1200, 1320]	[1320, 1440]
Recommended service time interval	[30, 120]	[30, 120]	[30, 120]	[30, 120]
Service venue capacity interval	[10, 200]	[10, 200]	[10, 200]	[10, 200]
Probability of generation	0.1	0.1	0.1	0.7

**Table 4:** Principal components and cumulative contribution rates.

Principal Components	Eigenvalue	Contribution Rate (%)	Cumulative Contribution Rate (%)
1	47.49907	52.2951%	52.2951%
2	23.19897	25.5414%	77.8366%
3	6.133428	6.7527%	84.5893%
4	5.092093	5.6063%	90.1955%
5	3.384269	3.7260%	93.9215%
6	2.924888	3.2202%	97.1418%
7	2.367477	2.6065%	99.7483%
8	0.226623	0.2495%	99.9978%

**Table 5:** The predictive performance comparisons of different models.

Model	RMSE	MAPE	MAE	$R^{2}$
ASFSSA-KELM	0.0024	1.09%	0.0017	0.9886
VPPSO-KELM	0.0036	1.69%	0.0027	0.9728
SSA-KELM	0.0031	1.40%	0.0022	0.9817
GWO-KELM	0.0042	1.95%	0.0031	0.9677
KELM	0.0097	4.81%	0.0075	0.8277
ELM	0.0092	4.56%	0.0071	0.8439
BP	0.0054	2.59%	0.0041	0.9462
LSTM	0.0076	3.55%	0.0055	0.8934

**Table 6:** Improvement in RMSE between different prediction models (%).

Model	ASFSSA-KELM	VPPSO-KELM	SSA-KELM	GWO-KELM	KELM	ELM	BP	LSTM
ASFSSA-KELM	0.00	32.94	20.46	41.70	75.00	73.66	55.13	68.19
VPPSO-KELM	−49.11	0.00	−18.60	13.06	62.72	60.72	33.09	52.57
SSA-KELM	−25.72	15.69	0.00	26.70	68.56	66.88	43.59	60.01
GWO-KELM	−71.52	−15.03	−36.43	0.00	57.11	54.82	23.04	45.44
KELM	−299.95	−168.21	−218.11	−133.18	0.00	−5.35	−79.45	−27.22
ELM	−279.63	−154.59	−201.96	−121.33	5.08	0.00	−70.34	−20.76
BP	−122.87	−49.46	−77.27	−29.94	44.28	41.29	0.00	29.11
LSTM	−214.38	−110.83	−150.06	−83.29	21.39	17.19	−41.06	0.00

**Table 7:** Improvement in MAPE between different prediction models (%).

Model	ASFSSA-KELM	VPPSO-KELM	SSA-KELM	GWO-KELM	KELM	ELM	BP	LSTM
ASFSSA-KELM	0.00	35.47	21.90	44.17	77.33	76.10	57.92	69.26
VPPSO-KELM	−54.97	0.00	−21.03	13.49	64.87	62.97	34.79	52.36
SSA-KELM	−28.04	17.38	0.00	28.52	70.97	69.40	46.12	60.64
GWO-KELM	−79.13	−15.59	−39.90	0.00	59.39	57.19	24.62	44.93
KELM	−341.10	−184.64	−244.51	−146.25	0.00	−5.41	−85.63	−35.62
ELM	−318.47	−170.03	−226.83	−133.61	5.13	0.00	−76.10	−28.66
BP	−137.63	−53.34	−85.59	−32.66	46.13	43.21	0.00	26.94
LSTM	−225.26	−109.89	−154.03	−81.58	26.26	22.27	−36.88	0.00

**Table 8:** Improvement in MAE between different prediction models (%).

Model	ASFSSA-KELM	VPPSO-KELM	SSA-KELM	GWO-KELM	KELM	ELM	BP	LSTM
ASFSSA-KELM	0.00	35.56	22.01	44.27	76.79	75.57	57.71	68.43
VPPSO-KELM	−55.19	0.00	−21.03	13.52	63.97	62.09	34.37	51.01
SSA-KELM	−28.23	17.37	0.00	28.54	70.23	68.68	45.78	59.52
GWO-KELM	−79.45	−15.63	−39.95	0.00	58.34	56.17	24.12	43.36
KELM	−330.78	−177.58	−235.95	−140.06	0.00	−5.23	−82.17	−35.98
ELM	−309.37	−163.79	−219.25	−128.13	4.97	0.00	−73.11	−29.22
BP	−136.48	−52.38	−84.42	−31.78	45.11	42.23	0.00	25.35
LSTM	−216.80	−104.14	−147.06	−76.54	26.46	22.61	−33.97	0.00

**Table 9:** Improvement in $R^{2}$ between different prediction models (%).

Model	ASFSSA-KELM	VPPSO-KELM	SSA-KELM	GWO-KELM	KELM	ELM	BP	LSTM
ASFSSA-KELM	0.00	1.62	0.69	2.15	19.43	17.14	4.48	10.65
VPPSO-KELM	−1.59	0.00	−0.91	0.53	17.53	15.28	2.81	8.89
SSA-KELM	−0.69	0.92	0.00	1.45	18.61	16.33	3.75	9.89
GWO-KELM	−2.11	−0.52	−1.43	0.00	16.91	14.67	2.27	8.32
KELM	−16.27	−14.92	−15.69	−14.47	0.00	−1.92	−12.52	−7.35
ELM	−14.63	−13.25	−14.04	−12.80	1.95	0.00	−10.81	−5.54
BP	−4.28	−2.74	−3.62	−2.22	14.31	12.12	0.00	5.91
LSTM	−9.62	−8.16	−9.00	−7.68	7.93	5.87	−5.58	0.00

**Table 10:** Pseudo code for determining the optimal number of task units.

Determination of the Optimal Number of Task Units
Input: Search range for number of task units $\left[2,\;\sqrt{M}\right]$
Output: Optimal number of task units $k_{opt}$
1: **for** k in $\left\{2,3,\ldots ,\sqrt{M}\right\}$ **do** 2: Randomly generate populations based on the value of k 3: Completion of task unit partition using the KPCA-ASFSSA-KELM-GBCA
4: Calculate the value of validity index based on the results of the partition of task units 5: **end for** 6: Sort *k*-values according to the values of the task unit partition validity indices
7: Output the optimal number of task units $k_{opt}$

**Table 11:** Performance comparison of GBCA and other well-known clustering algorithms.

Instance	Validity Index	GBCA	ASFSSA	BWO	PSO	K-Means	K-Medoids	SC	K-Means++
Instance 1	APB	−0.1661	−0.1641	−0.1631	−0.1639	−0.1647	−0.1647	−0.1647	−0.1647
	${RR}_{s}$	0.0194	0.0824	0.0543	0.2477	0.0543	0.1437	0.0492	0.0507
	${RR}_{v}$	0.0309	0.0343	0.0424	0.0408	0.0765	0.0926	0.0645	0.0641
	F	0.0389	0.1240	0.1454	0.3285	0.3483	0.5348	0.2715	0.2707
	SD(F)	0.0021	0.0085	0.0092	0.0153	0.0420	0.0512	0.0187	0.0179
Instance 2	APB	−0.1624	−0.1599	−0.1602	−0.1602	−0.1601	−0.1595	−0.1598	−0.1598
	${RR}_{s}$	0.0307	0.1100	0.1172	0.2040	0.0482	0.1098	0.0377	0.0473
	${RR}_{v}$	0.0399	0.0449	0.0555	0.0547	0.0609	0.0986	0.0644	0.0633
	F	0.1074	0.2196	0.2898	0.3721	0.2532	0.5417	0.2641	0.2673
	SD(F)	0.0054	0.0132	0.0188	0.0260	0.0203	0.0488	0.0211	0.0214
Instance 3	APB	−0.1634	−0.1622	−0.1618	−0.1620	−0.1624	−0.1627	−0.1626	−0.1624
	${RR}_{s}$	0.0420	0.1443	0.1353	0.1411	0.0509	0.0845	0.0468	0.0508
	${RR}_{v}$	0.0467	0.0535	0.0646	0.0600	0.0710	0.0828	0.0783	0.0761
	F	0.1587	0.3032	0.3609	0.3392	0.3143	0.4187	0.3539	0.3452
	SD(F)	0.0078	0.0232	0.0315	0.0283	0.0247	0.0412	0.0305	0.0292
Instance 4	APB	−0.1658	−0.1656	−0.1660	−0.1661	−0.1652	−0.1653	−0.1652	−0.1652
	${RR}_{s}$	0.0302	0.1149	0.0924	0.1264	0.0412	0.1157	0.0453	0.0437
	${RR}_{v}$	0.0484	0.0518	0.0656	0.0554	0.0782	0.0823	0.0752	0.0745
	F	0.1545	0.2602	0.3198	0.2926	0.3451	0.4442	0.3311	0.3254
	SD(F)	0.0076	0.0179	0.0252	0.0230	0.0288	0.0393	0.0276	0.0272

**Table 12:** Improvements in the clustering effectiveness indicator of the proposed algorithm over other algorithms (%).

Instance	ASFSSA	BWO	PSO	K-Means	K-Medoids	SC	K-Means++	Average
Instance 1	68.64	73.25	88.16	88.83	92.73	85.68	85.63	83.27
Instance 2	51.08	62.92	71.12	57.56	80.17	59.32	59.80	63.14
Instance 3	47.66	56.02	53.21	49.50	62.09	55.15	54.02	53.95
Instance 4	40.63	51.69	47.21	55.24	65.22	53.35	52.53	52.27
Average	52.00	60.97	64.93	62.78	75.05	63.38	63.00	63.16

**Table 13:** Improvements in APB of the proposed algorithm over other algorithms (%).

Instance	ASFSSA	BWO	PSO	K-Means	K-Medoids	SC	K-Means++	Average
Instance 1	1.24	1.88	1.34	0.88	0.90	0.89	0.89	1.15
Instance 2	1.57	1.41	1.42	1.44	1.88	1.68	1.65	1.58
Instance 3	0.71	0.99	0.86	0.57	0.39	0.48	0.61	0.66
Instance 4	0.13	−0.13	−0.14	0.41	0.32	0.36	0.40	0.19
Average	0.91	1.04	0.87	0.83	0.87	0.85	0.89	0.89

**Table 14:** Improvements in ${RR}_{s}$ of the proposed algorithm over other algorithms (%).

Instance	ASFSSA	BWO	PSO	K-Means	K-Medoids	SC	K-Means++	Average
Instance 1	76.44	64.24	92.17	64.24	86.50	60.53	61.70	72.26
Instance 2	72.13	73.85	84.97	36.36	72.08	18.74	35.23	56.19
Instance 3	70.91	68.98	70.25	17.47	50.31	10.23	17.29	43.63
Instance 4	73.73	67.32	76.11	26.74	73.92	33.34	30.92	54.58
Average	73.30	68.60	80.88	36.20	70.70	30.71	36.29	56.67

**Table 15:** Improvements in ${RR}_{v}$ of the proposed algorithm over other algorithms (%).

Instance	ASFSSA	BWO	PSO	K-Means	K-Medoids	SC	K-Means++	Average
Instance 1	9.80	26.98	24.16	59.53	66.60	52.04	51.75	41.55
Instance 2	11.26	28.09	27.12	34.48	59.55	38.04	37.00	33.65
Instance 3	12.78	27.68	22.21	34.23	43.63	40.37	38.68	31.37
Instance 4	6.69	26.26	12.70	38.15	41.24	35.68	35.08	27.97
Average	10.13	27.25	21.55	41.60	52.76	41.53	40.63	33.64

## Data Availability

The data presented in this study are available on request from the corresponding author.
